# A Multivariate Approach to Determine the Dimensionality of Human Facial Asymmetry

**DOI:** 10.3390/sym12030348

**Published:** 2020-03-01

**Authors:** Omid Ekrami, Peter Claes, Julie D. White, Seth M. Weinberg, Mary L. Marazita, Susan Walsh, Mark D. Shriver, Stefan Van Dongen

**Affiliations:** 1 Evolutionary Ecology Group, Department of Biology, University of Antwerp, 2610 Antwerp, Belgium; stefan.; 2 Department of Electrical Engineering, ESAT/PSI, KU Leuven, 3000 Leuven, Belgium;; 3 Medical Imaging Research Center, UZ Leuven, 3000 Leuven, Belgium; 4 Department of Human Genetics, KU Leuven, 3000 Leuven, Belgium; 5 Department of Anthropology, Pennsylvania State University, University Park, State College, PA 16801, USA;; 6 Department of Oral Biology, Center for Craniofacial and Dental Genetics, University of Pittsburgh, Pittsburgh, PA 15260, USA;; 7 Department of Human Genetics, Graduate School of Public Health, University of Pittsburgh, Pittsburgh, PA 15260, USA; 8 Department of Biology, Indiana University Purdue University Indianapolis (IUPUI), Indianapolis, IN 46202, USA;

**Keywords:** fluctuating asymmetry, 3D morphometrics, directional asymmetry, sexual dimorphism

## Abstract

Many studies have suggested that developmental instability (DI) could lead to asymmetric development, otherwise known as fluctuating asymmetry (FA). Several attempts to unravel the biological meaning of FA have been made, yet the main step in estimating FA is to remove the effects of directional asymmetry (DA), which is defined as the average bilateral asymmetry at the population level. Here, we demonstrate in a multivariate context that the conventional method of DA correction does not adequately compensate for the effects of DA in other dimensions of asymmetry. This appears to be due to the presence of between-individual variation along the DA dimension. Consequently, we propose to decompose asymmetry into its different orthogonal dimensions, where we introduce a new measure of asymmetry, namely fluctuating directional asymmetry (F-DA). This measure describes individual variation in the dimension of DA, and can be used to adequately correct the asymmetry measurements for the presence of DA. We provide evidence that this measure can be useful in disentangling the different dimensions of asymmetry, and further studies on this measure can provide valuable insight into the underlying biological processes leading to these different asymmetry dimensions.

## 1. Introduction

Bilateral symmetry, in which the left and right side of an organism are mirrored replicates of each other, is a prominent feature of most animals. Nevertheless, the ideal state of perfect symmetry is rare in nature, and deviations, known as asymmetries, are ubiquitous at both the individual and population level. Since both sides of an organism reflect replicates of the same developmental event, asymmetric development points towards the presence of developmental noise, as well as the body’s inability in buffering the noise [1]. Based on the population distribution of asymmetry, three types are traditionally recognized [2]: directional asymmetry (DA); anti-symmetry (AS); and fluctuating asymmetry (FA). DA occurs when the average asymmetry in a population is *not* zero; this means that the population shows a systematic difference in the development of the structure of interest, such that either the left or the right is more or less prominent. AS refers to a situation where the average of the left–right distribution is zero, but the distribution shows bimodality or some degree of platykurtosis (a flat distribution). Such a situation indicates the presence of DA in a population, but with alternating prominent sides. FA is defined as directionally random deviations from perfect symmetry around a zero mean, or around DA (or AS) if present. As these three types of asymmetry are not mutually exclusive [2], the signed left–right differences—which from now on will be referred to as total asymmetry (TA)—can be a combination of any of these three types (Table 1).

Fluctuating asymmetry in human faces has been extensively studied in an evolutionary context [3–5]. It has often been argued that FA is the outcome of random perturbations in developmental processes, and can be an honest marker of biological fitness [6–7], as well as an indicator of environmental and genetic stress caused by directional selection [8,9]. It is traditionally considered to be a measure of developmental instability (DI), i.e., the inability of an organism to block out the genetic and environmental stresses that tend to affect its development towards a consistent phenotype [1]. It should be noted, however, that there is still a lot of debate about the links between FA, DI and measures of stress and fitness. On the other hand, DA (or AS) is considered to be attributable to genetic components [10–12] or environmental differences between sides (e.g., biomechanical pressures and handedness) [13–15]. With a few exceptions, DA is not considered to be related to DI (Lens & Van Dongen, 2008). In nearly all studies, DA is described as the average asymmetry across the population. This is considered a statistical nuisance when estimating DI, and by subtracting DA from the individual total asymmetries (TA), FA is obtained for each individual. A critical assumption herewith is that DA, or rather the developmental processes that lead to it, are identical in all individuals. There are, however, several lines of evidence that question the validity of this assumption. Differences in biomechanical pressures between left and right limbs (affected by symmetric and asymmetric activities) lead to different magnitudes of TA [16]. Furthermore, Stige et al. [17] have demonstrated the presence of heterogeneity in DA among individuals, as well as a residual correlation between DA and FA even after correcting for DA. Klingenberg (2003) also criticized simple corrections for DA, stating that it could only be meaningful in cases of small DA. Furthermore, it is important to note that asymmetry measures at population and individual levels could reflect different underlying evolutionary mechanisms. Therefore, examining DA at the individual level can be beneficiary in many studies. There is thus a clear need for greater insight into the biological relevance of DA and its potential variation among individuals. One of the main aims of this study is to be able to quantify this variation in the direction of DA. In order to do so, however, a starting point is to quantify different dimensions and patterns of asymmetry.

**Figure 1.**
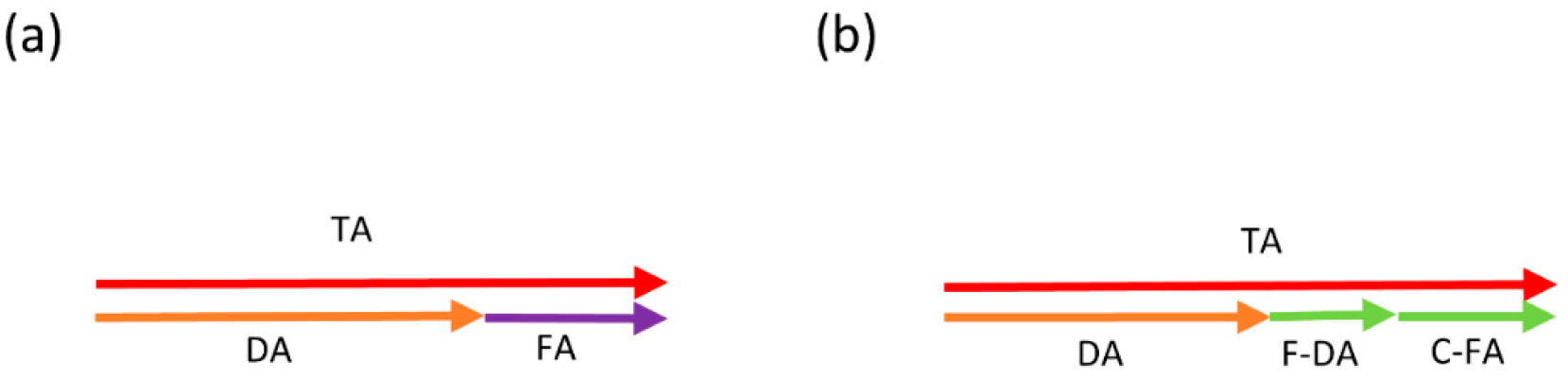
Representation of linear measurements commonly used for asymmetry analysis. (**a**) There is no variation between the individuals in the DA direction, therefore FA and DA vectors are not correlated. In this scenario, FA can be adequately calculated by only removing the effect of DA; (**b**) in case the FA vector is correlated with the DA, its component in the direction of DA (F-DA) and the orthogonal component (C-FA) cannot be effectively separated.

**Figure 2.**
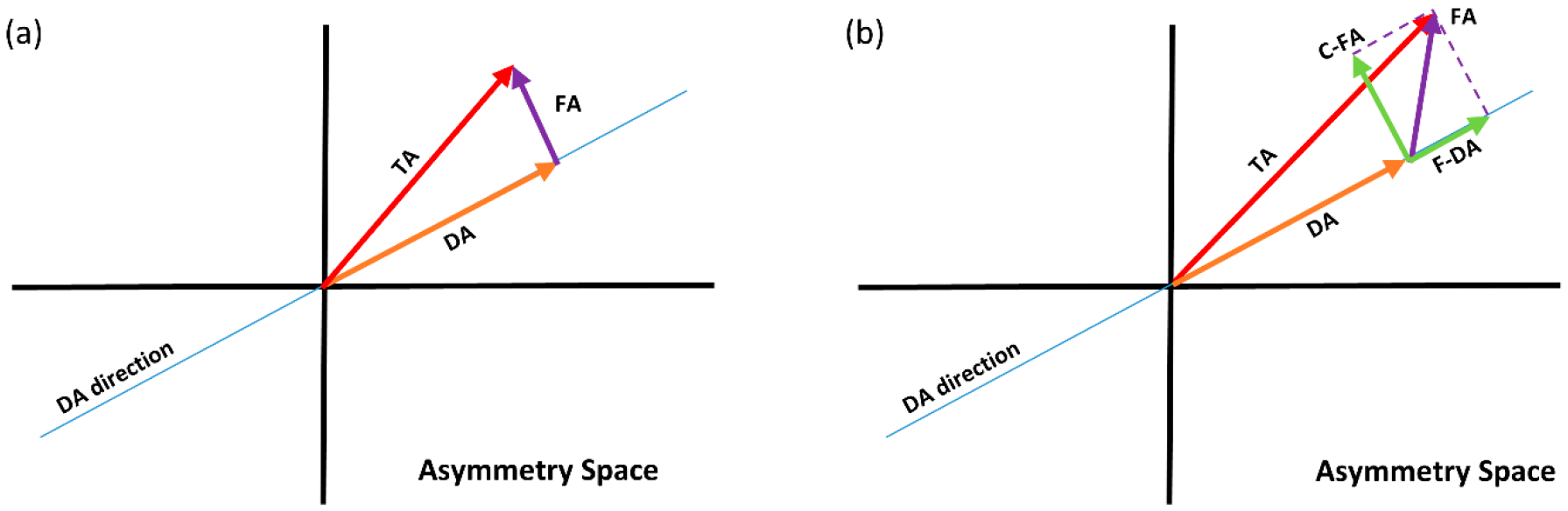
A 2D representation of the asymmetry space. The plot on the left (**a**) represents a case where the calculated FA vectors are orthogonal to the DA vector. The plot on the right (**b**) shows a scenario where the calculated FA vector has a component parallel to the DA vector.

In order to be able to study individual variation in the three different components of TA (i.e., FA, DA and AS) and how they relate to measures of genetic and environmental stress, it is crucial to develop a technique to adequately obtain estimates of individual FA that are unrelated to all variation in the dimension of DA. Notably, this is simply not possible for linear measurements of traits (e.g., digit length, wrist circumference, etc.). To illustrate this, suppose that the wrist circumference of individual X equals 21 cm and 22 cm on the left and right side respectively. In the total sample, the average TA (right minus left) equals 0.5 (i.e., DA). Traditionally, one would calculate FA as *(R* − *L)* – *DA* = *(22 − 21)* *− 0.5* = *0.5*. If the population of interest was completely homogenous (regarding genetic variation and biomechanical pressure), meaning no variation in DA among individuals (Figure 1a), then this would be a reasonable estimate of fluctuating asymmetry. However, in studies of asymmetry in humans or animals, it is a rather impractical task to assure the complete homogeneity of the population. Therefore, if, for example, DA emerges due to handedness, it might be possible that individual X, who, for example, plays tennis intensively, has a higher level of right-biased asymmetry due to the significant differences in biomechanical loading between the two sides. In this case, the DA-corrected asymmetry of 0.5 is composed of two components: the additional DA due to the higher differential (0.75 − 0.5 = 0.25); and the asymmetry due to random perturbations (0.25). Obviously, in a one-dimensional setting, these two components cannot be separated from each other morphologically (Figure 1b). Because these components of asymmetry around DA are both variations among individuals, we refer to the component in the direction of average asymmetry (DA) as *fluctuating directional asymmetry* (F-DA), and the individual variation due to DI as the *corrected FA* (C-FA). To effectively disentangle F-DA and C-FA, measurements in two or more dimensions are required (e.g., the coordinates of biological landmarks), such that both sources of individual variation can be estimated separately. The most common way to do that statistically is to use orthogonal dimensions. We can see this in Figure 2, a 2D equivalent of Figure 1, where in (a) the FA vector is perpendicular to the DA vector, as opposed to (b) where FA has a component in the direction of DA. What is crucial, and highlighted in Figure 2b, is that for the estimation of C-FA, an adequate correction for both DA and F-DA is required. It is important to highlight here that this will lead to uncorrelated asymmetry dimensions, yet it does not necessarily imply that the biological or developmental processes leading to the actual asymmetries of the different individuals are independent across these dimensions, an aspect that will be studied explicitly here (see below).

**Figure 3.**
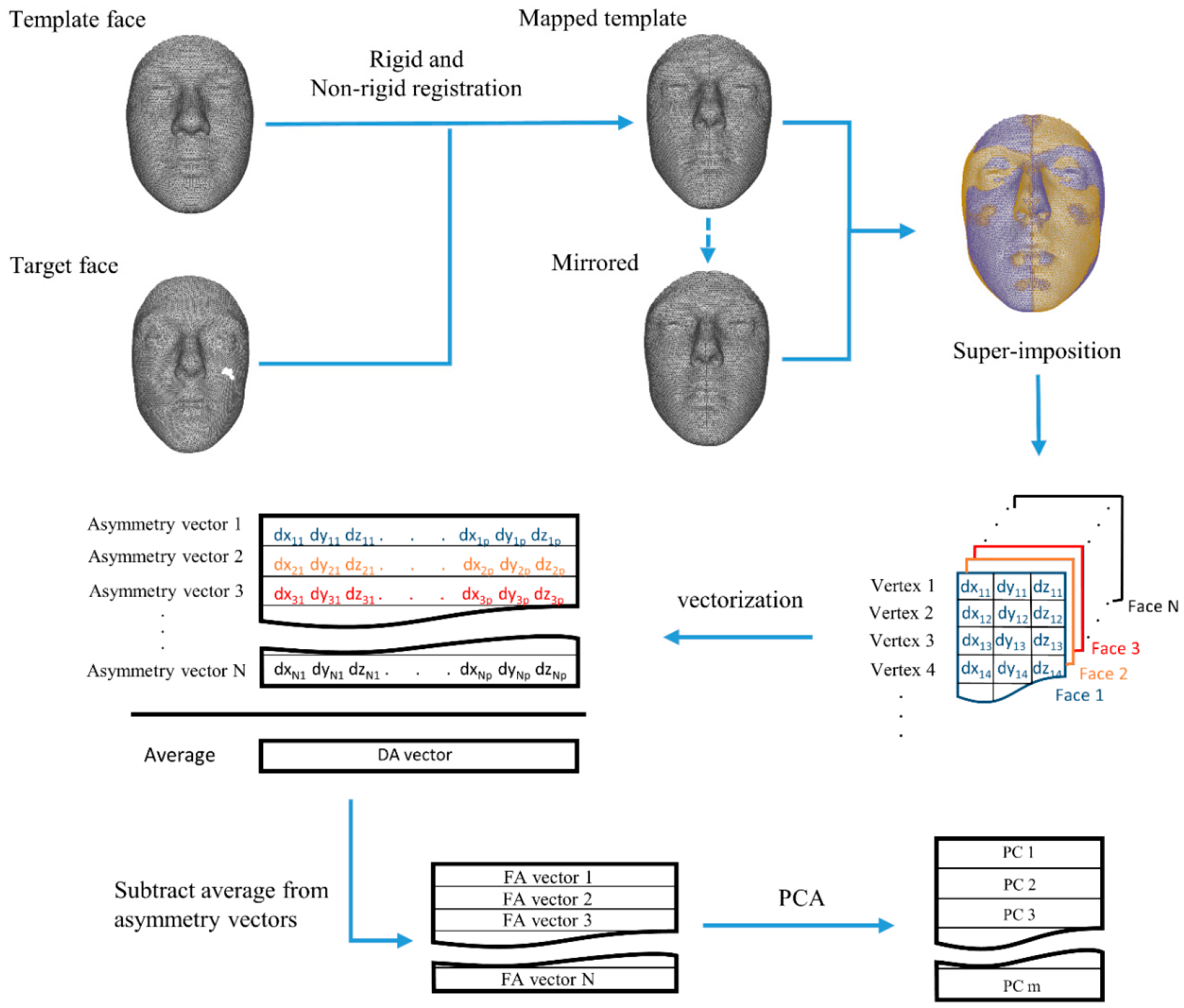
Flow chart of the algorithm used to obtain FA vectors. The template face is mapped onto each face, and the reflection is obtained. Each face and its mirror are superimposed and subtracted, and the subtraction matrix is vectorized to obtain the matrix of asymmetry vectors (TA). The average of these vectors is considered as DA, and by removing the DA from each row of the matrix, the FA vectors are calculated. Different dimensions of FA can then be explored by applying a PCA on the vectors.

In this study, we attempt to decompose asymmetry into different dimensions. We developed a multivariate method to capture individual variations in the direction of DA, and use them to extract measures of FA that are structurally and statistically free from DA dimension. This approach provides opportunities to study the biological relevance of the different asymmetry dimensions, and may shed light on the developmental processes that lead to them. We analyzed asymmetry in a large cohort of faces from persons of recent European–American ancestry in a multivariate setting using spatially dense 3D scans (Figure 3). The faces were then transferred to a standardized and spatially dense setting, and the asymmetry vectors (TA) were calculated following Ekrami et al. (2018). First, we determine the population level DA separately for males and females, study the correspondence, and test for a statistically significant difference between both sexes. We then correct TA for DA to obtain FA vectors, initially ignoring possible variation in asymmetry along the dimension of DA (i.e., ignoring the presence of F-DA). This approach is conceptually identical to correcting for DA in linear measurements, and will allow us to show that even after this correction, the FA vectors are correlated with the DA vectors, and that this ‘naive’ approach results in individual FA measures that contain variation in the dimension of DA. We then apply our newly developed approach (i.e., correcting TA for F-DA, using a regression technique similar to methods applied to correct shapes for allometric effects, where C-FA is calculated as the residual of regression of TA over DA; see details below) to disentangle the individual variation in asymmetry around DA into orthogonal dimensions of F-DA and C-FA. Next, we describe the patterns in asymmetry and discuss differences between males and females. Finally, we explore correlations between the absolute values of individual scores of C-FA and F-DA. This allows us to investigate the extent and magnitude of individual asymmetry variation in the dimension of DA (F-DA), as well as possible similarities with the orthogonal dimensions (C-FA). We thus provide a first attempt to estimate the extent to which similar underlying developmental processes lead to higher asymmetry along the different asymmetry dimensions, or whether they contain independent developmental information. Further research will inevitably be required to evaluate associations of the asymmetry dimensions and measures of health, fitness and quality, as well as to explore their genetic architecture.

## 2. Materials and Methods

The dataset comprises 1246 adult face scans (623 males and 623 females) of people between the ages of 18 and 35, with a BMI within the normal range (18.5–24.9 kg/m^2^), all with self-reported European-derived ancestry and no history of any significant facial trauma or facial surgery, or any medical condition that might alter facial structure. The facial scans originated from a mixture of several studies at the University of Pittsburgh, Pennsylvania State University and Indiana University–Purdue University, Indianapolis. The scans were made using two stereophotogrammetry systems: the VECTRA H1 camera (Canfield Scientific, Parsippany, NJ, USA); and the 3dMDface system (3dMD, Atlanta, GA, USA).

The total asymmetry (TA) was determined using a multivariate spatially dense approach [18,19]. Briefly, a symmetrical template face comprised of n = 7160 densely located paired vertices was mapped onto each of the faces in the dataset, using a non-rigid iterative closest points (ICP) algorithm. The reflection of the faces was obtained by reversing the sign of the X coordinates of the vertices (the x-axis passes through the middle of the face), and then relabeling the vertices with their paired counterparts in order to regain compatibility of the original face and its mirror [20]. The original faces and their reflections were then fed into a weighted generalized Procrustes analysis, in which a higher weight was assigned to the more symmetrical regions of the face [21]. The asymmetry vector (TA) for each face was then simply calculated by subtracting the mirrored face from the original. From this point on, the male and female faces were treated as two separate groups. Following the conventional approach, DA was calculated as a vector reflecting the average of TA (the asymmetry vectors) of all the faces in men and women (Figure 3). The significance of the acquired DA vectors (whether the average TA is different than zero), as well as sexual dimorphism in DA (the difference between DA in males and females), was tested, as described in detail by Claes et al. (2012). The DA was calculated as the difference in the sample mean of the original and the reflected faces, treated as different populations. Permutation tests were used on simple Euclidean distances (D-statistics) and Goodall’s F-test (F-statistics) to measure the significance of DA in both groups. The sexual dimorphism in DA was tested as the difference in the sample mean of TA matrices for men and women, and the significance was again checked using permutation tests.

Initially, we corrected the TA traditionally by subtracting the DA vector from each individual asymmetry vector. This approach ignores the between-individual asymmetry variation in the direction of DA (Figure 2a). We then used principal component analysis (PCA) to investigate the different dimensions of the FA vectors, and tested if these PCs correlate with the DA vectors in both men and women. This test evaluates if the FA dimensions contain any shared information with the dimension of DA, which ideally should not exist.

As we anticipated, there were significant correlations (see below), meaning that the FAs were not independent of variation in the dimension of DA. We thus propose to disentangle the components of asymmetry by obtaining individual variation in the dimension of DA (i.e., the F-DA vector) by regressing the FA vectors onto the DA vector. Following the principles of vector projection and the formula for an inner product of two vectors, the F-DA vector can then be calculated as

(1) $$\vec{FA}.\vec{DA}=|\vec{FA}||\vec{DA}|\;\text{cos}\alpha$$

(2) $$|\vec{F-DA}|=|\vec{FA}|\;\text{cos}\alpha =\frac{\vec{FA}.\vec{DA}}{\left|\vec{DA}\right|}$$

(3) $$\vec{F-DA}=\frac{\vec{FA}.\vec{DA}}{\left|\vec{DA}\right|}\frac{\vec{DA}}{\left|\vec{DA}\right|}=\frac{\vec{FA}.\vec{DA}}{|\vec{DA}{|}^{2}}\vec{DA}$$

where $\vec{FA}.\vec{DA}$ is the dot product of the FA and DA vectors, *α* is the angle between these two vectors and $\frac{\vec{DA}}{\left|\vec{DA}\right|}$ is the unit vector representing the direction of DA. The term $\frac{\vec{FA}.\vec{DA}}{\left|\vec{DA}\right|}-|\vec{DA}|$ also gives us the signed magnitude of F-DA. This magnitude represents individual variation in asymmetry in the direction of DA, and can be used to check for the presence of anti-symmetry by analyzing the distribution of the F-DA magnitudes. This approach is based on the definition of AS, which occurs by mixing two genotypes, with each having directional asymmetry in opposite directions [12]. Between-individual variation in asymmetry corrected for both DA and F-DA (i.e., the C-FA vector) can then be obtained by subtracting F-DA from the FA vector. This process follows a similar concept to that of correcting allometry [22]. We applied PCA on the acquired C-FA vectors, and checked the PCs for correlations with the DA vector, in order to validate our expectation of orthogonality (i.e., no correlation) for these vectors. As noted above, this analysis does not test for similarities in the developmental processes leading to the morphological asymmetries, but only checks if these dimensions are indeed orthogonal. These PCs were then used to explore and compare the different dimensions of C-FA. We also analyzed the distribution of F-DA magnitudes, and compared them between men and women. The kurtosis of the distributions of F-DA was calculated to check for the possible presence of AS. Finally, on an individual basis, the absolute values of the individual PC scores of C-FA and of F-DA were correlated to explore evidence for joint underlying developmental processes.

## 3. Results

### 3.1. Facial Directional Asymmetry in Men and Women

**Figure 4.**
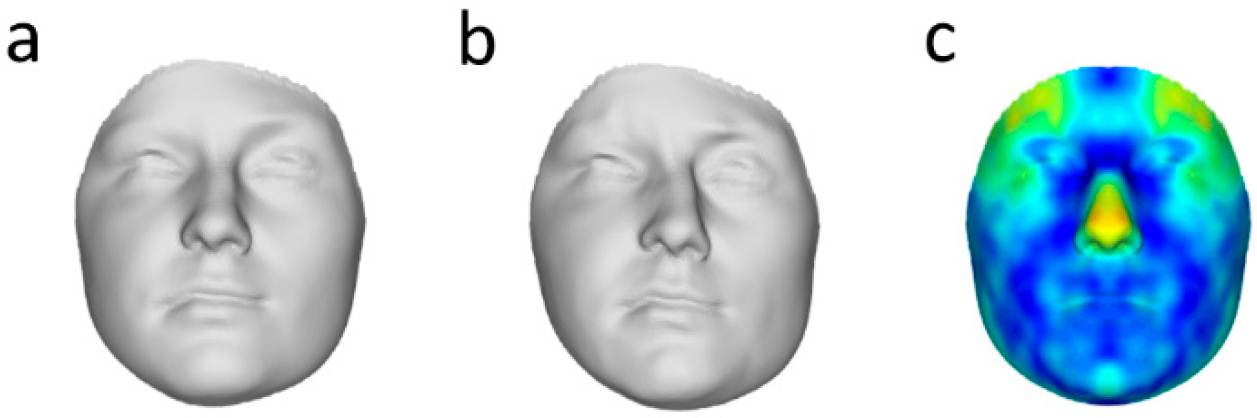
(**a**) Female and (**b**) male directional asymmetry, amplified 10 times and visualized onto the average face; and (**c**) heat-map of the difference between DA in both sexes. The values are scaled (amplified) equally in all three figures for visual purposes.

Both directional asymmetry and the sexual dimorphism in DA were statistically significant (DA men: D = 21.5, *p* = 0.001; DA women: D = 15.0, *p* = 0.01; sexual dimorphism in DA: D = 14.0, *p* = 0.003). Tests based on F-statistics confirmed these conclusions (details not shown). The visualization of DA in men and women, as well as a heat-map of the DA differences, are shown in Figure 4.

**Figure 5.**
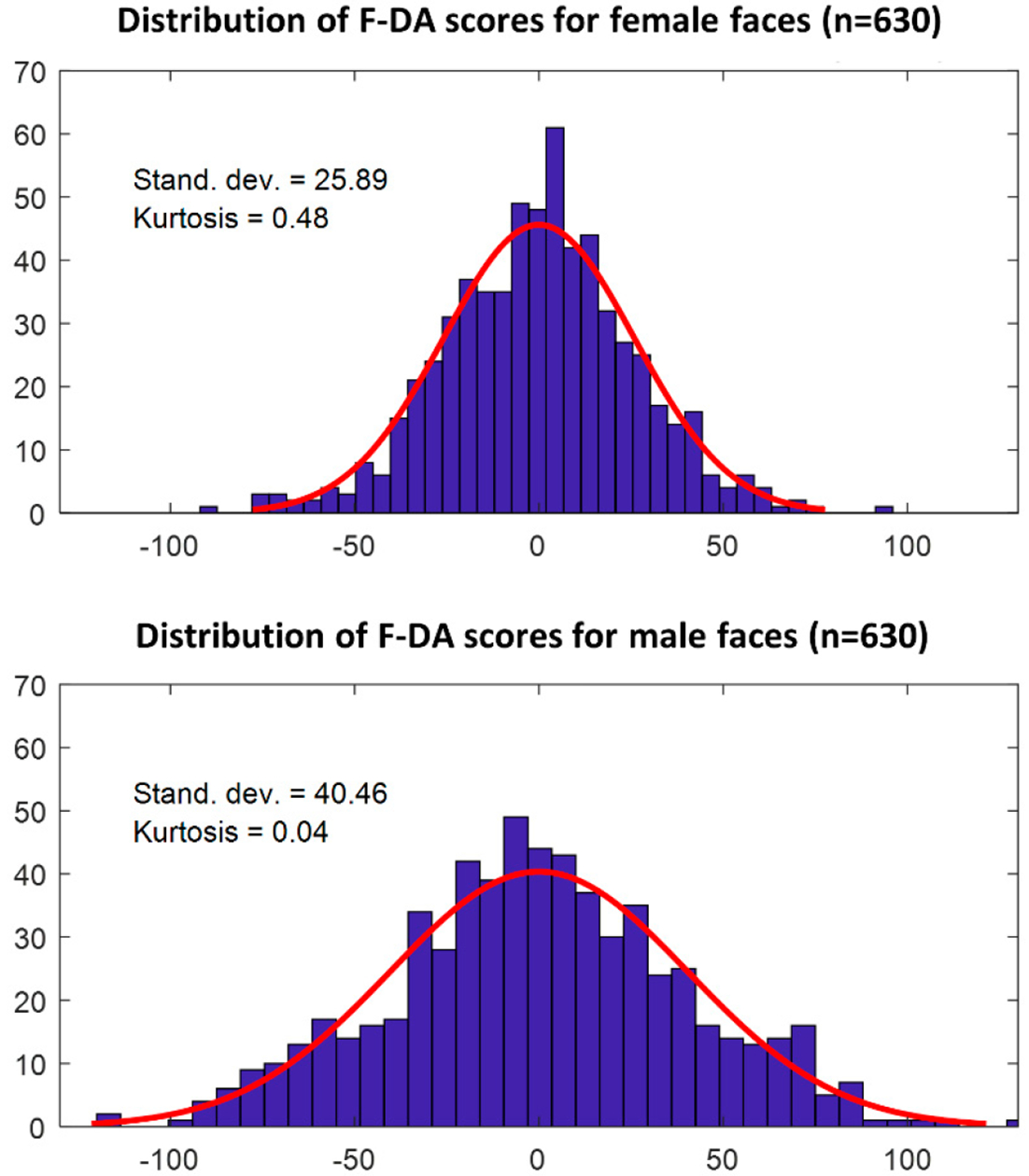
Histogram of the magnitudes of F-DA vectors for females (**above**) and males (**below**). The kurtosis measurement validates the normality of the distributions. This also suggests a lack of AS in the faces. Based on the standard deviations, we can see that the male subgroup shows a higher effect of DA on individual faces.

At the individual level, the F-DA vectors reflect individual variation in asymmetry in the direction of DA in men and women. The distribution of signed magnitudes of the F-DA vectors (the F-DA scores, Figure 5) did not show any signs of platykurtosis or a bimodal pattern; this supports the absence of AS. Histograms of the signed magnitude of the calculated F-DA vectors are presented in Figure 5. Men showed a significantly higher variance, and thus had magnitudes of variation in F-DA scores among individuals when compared to women (F_622,622_ = 0.40, *p* < 0.001).

### 3.2. Estimating Fluctuating Asymmetry

Following the conventional method of DA correction—i.e., simply subtracting the average asymmetry from TA (Figure 2a)—the principal components of the FA vectors were obtained, and their correlation with the DA vector was calculated for the first five PCs (Table 2). The first five PCs of FA, representing the main directions of variation in FA, explained 62% and 60% of the asymmetry variation in men and women respectively (Table 2). Many of these dimensions correlated significantly with the dimension of the DA vector in both men and women, where correlations were stronger in men (Table 2).

The same set of correlations was calculated after performing the F-DA correction, i.e., removing between-individual variation in the direction of DA. As expected, since the DA and C-FA vectors are by definition orthogonal, there was no correlation observed between these vectors (r < 0.001, *p* > 0.1 for all PCs), demonstrating that the C-FA vectors are adequately corrected for all asymmetry variations in the direction of DA.

**Figure 6.**
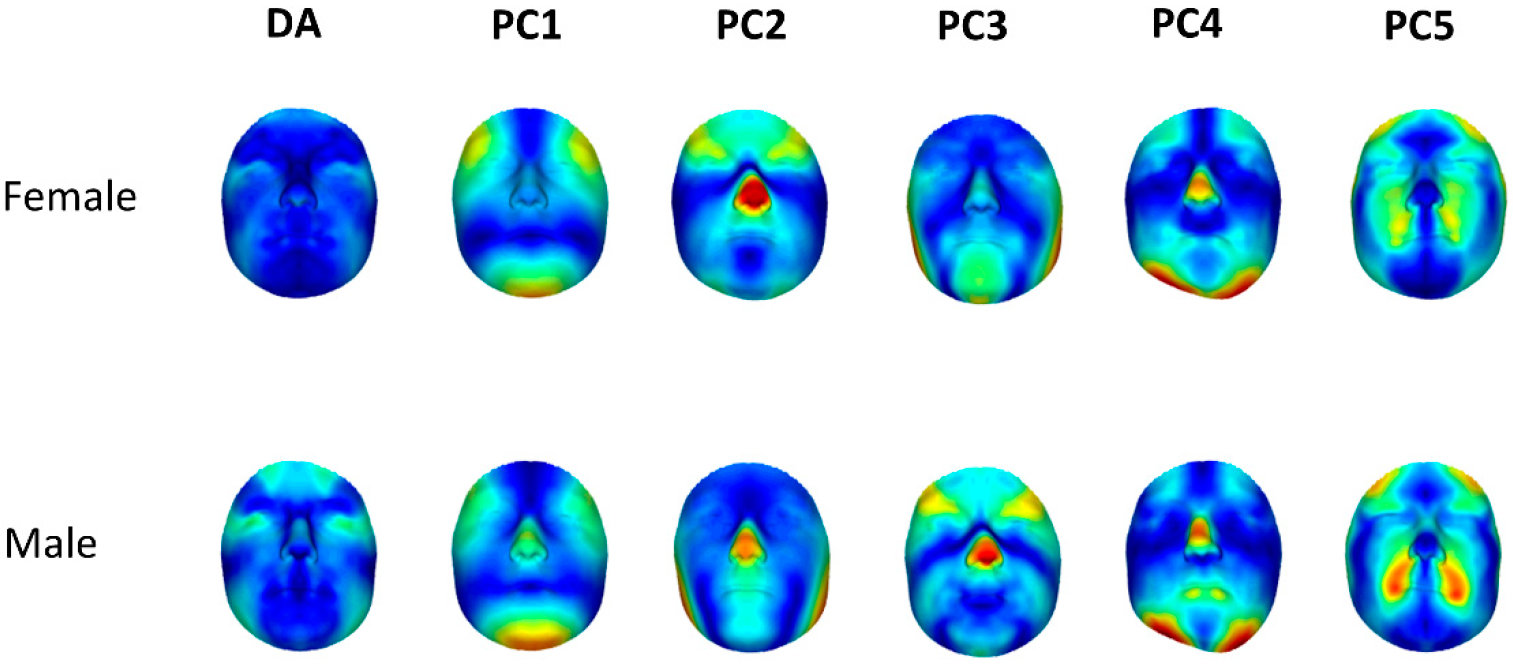
Heat map of DA and the first 5 PCs of C-FA for both males and females. The differences and similarities between the two groups are clear in this figure. The variations are scaled (amplified) for visual purposes. The blue color on the heat maps shows low variations, and the red regions correspond to high variations. The same scale has been used for both groups.

Figure 6 presents heat maps of the asymmetry dimensions (the DA vector, as well as the first five PCs of C-FA) in men and women. We can visually confirm that DA is more prominent in men. A similarity of patterns is observed across the different dimensions of C-FA between men and women, although contrary to DA, the expression of asymmetry in different dimensions does not seem to be more prominent in either sex.

### 3.3. Correlations between C-FA and F-DA Scores

If the same underlying developmental processes would lead to between-individual differences in asymmetry in the different dimensions identified here, the magnitudes of the scores (i.e., the absolute values) are expected to correlate positively. The absolute values of the C-FA scores from the five PCs did not correlate significantly among themselves, neither in men nor women (all r < 0.001). Correlations between the absolute values of the F-DA and those of C-FA were somewhat higher, and statistically significant in several cases (Table 3), with the highest correlation between |F-DA| and |C-FA| of PC3 in men (Table 3), suggesting that some of the between-individual variations in these two dimensions have a shared biological background.

## 4. Discussions

Fluctuating asymmetry is commonly used as an indicator of developmental instability. The presence of directional asymmetry, however, biases the measurements of FA [11], and should be accounted for. Subtracting population-level DA from all individual asymmetry values can only provide unbiased FA measures (possibly reflecting DI) if the underlying DA is the same (both in magnitude and direction) for all individuals. However, some studies have suggested the presence of individual-level variability in DA [1,17]. While measurements in one dimension (e.g., length, circumference) do not allow differentiation between variation in DA and FA dimensions among individuals, it is possible to identify these different dimensions in 2D or 3D datasets. We developed a method to define an asymmetry dimension in the direction of population-level average asymmetry (DA), and to correct for between-individual variation in this dimension. Consequently, the new measures of individual FA generated are structurally free of any variation in DA direction.

We apply this technique to a large set of human facial 3D scans. The presence of statistically significant DA was detected in both men and women, and the degree of DA was more prominent in males. This sexual dimorphism is in line with the findings of Claes et al. (2012), but in contrast to those reported by Ercan et al. (2008), who found female faces to show higher DA. This might be due to a difference in methodology, as in our study, as well as Claes et al. (2012), spatially dense 3D scans were used, while Ercan et al. (2008) used linear distances for a much smaller number of landmarks. Indeed, the use of a limited number of landmarks in two dimensions to measure asymmetry may also lead to a loss of useful information in under-sampled areas or the unused dimension (Ekrami et al., 2018). In our analyses, differences in DA between men and women were primarily present in the temporal frontal region of the face—both above and on the lateral sides of the eyes—which is a region that is generally under-represented in studies that use a limited number of landmarks (Figure 4c). This again highlights the advantages of using spatially dense 3D scans for studies of asymmetry.

Next to sexual differences in the amount of DA (i.e., the Euclidian distance), the morphological dimensions of DA differ as well. A test of correlation between the DA vectors, which reflect the dimension of DA, showed that they share about 60% of their information (R^2^ = 0.58). Therefore, about 40% of the average asymmetry is not shared between men and women (Figure 4). In addition, the amount of between-individual variation in this morphological direction is larger in men (Figure 5). Clearly, if one wants to generate FA estimates from human faces, corrections for DA should be done separately for men and women, and both the average directional asymmetry, as well as the between-individual variation in DA (which we termed F-DA), should be taken into account.

Indeed, simply subtracting the average DA to obtain an FA shape space still resulted in FA dimensions that correlate with the average DA. Regressing the DA-corrected asymmetries (conventional FA vectors) onto the DA vectors, obtaining the F-DA vectors and then subtracting the F-DA vectors from the FA vectors resulted in new asymmetry dimensions (C-FA). These were, as expected, completely uncorrelated with the dimension of DA in both men and women. We constructed orthogonal (i.e., directionally uncorrelated) dimensions, one of which reflects individual variation in the direction of DA, and the other reflects asymmetry variation in dimensions other than DA. These C-FA dimensions may be a more accurate indicator of DI. At this point, our approach to defining independent/orthogonal morphological dimensions reflecting DA and FA is purely technical. The next step is to evaluate what, if any, biological meaning these dimensions provide, which will require further study. We can nevertheless explore the actual asymmetry dimensions. Based on the asymmetry heat-maps, the first interesting result is the similarity of directions of FA between men and women. Similar to the findings of Claes et al. (2012), the first and most important dimension of FA is consistent with Enlow’s Counterpart Principle [23], where a strong asymmetry in the chin is balanced by a countering opposite asymmetry in the brow ridge and lower forehead. Furthermore, the third PC in the female cohort and the second PC in the male cohort show asymmetry in the gonion (or the angle of mandible) region, which has been reported to show high asymmetry in previous studies [24,25].

We can further investigate to what extent F-DA and the C-FA dimensions reflect mutual underlying developmental processes. Even though these dimensions are orthogonal, and thus uncorrelated in terms of their directions, the amount of asymmetry observed may be correlated. More specifically, if an individual has a high degree of asymmetry in the DA dimension (i.e., a relative extreme score of F-DA) and in other C-FA dimensions (relative extreme score for some of the PCs), this could hint towards a common developmental origin, with potentially shared developmental pathways related to DI. Broadly, our results indicated very small correlations among the individual scores in all dimensions (either between the PCs and F-DA or among the PCs themselves), except for the scores of the third PC of the male cohort, which show significant correlations with the F-DA scores. This means that the underlying developmental processes do not influence all dimensions similarly, which confirms the absence of an individual asymmetry parameter (IAP), as found in many studies [26–28]. Thus, since asymmetry develops largely independently in the different dimensions identified here, our results and methodology strengthen the need to approach studies of FA in a multivariate context, as opposed to calculating simple average multivariate asymmetries.

We conclude that measuring morphological asymmetry is less straightforward than previously thought. A simple measure of average left–right differences, either univariate or using 2D or 3D landmarks, cannot fully capture all dimensions of asymmetry. The conventional method of correcting FA for the effects of DA does not completely disentangle FA and DA, and further correction steps are required to partition total asymmetry into its various forming components. Using a 3D spatially dense approach [18], we can investigate the asymmetry space more thoroughly. We showed that asymmetry emerges in a non-uniform manner in different dimensions, and its magnitude and directions vary between the sexes. Therefore, we suggest that studies of asymmetry should be carried out using a multivariate framework, and males and females should be treated separately. The novel asymmetry measure of F-DA proved to be useful in disentangling different dimensions of asymmetry, although its origin remains unknown, and determining the biological meanings behind it requires further research. Future studies on the underlying genetic architecture and environmental exposure contributing to different dimensions of asymmetry will shed more light on the found correlations, as well as the underlying processes leading to them.

## Figures and Tables

**Table 1.** Description of abbreviations.

Abbreviation	Full Name	Description
**DA**	Directional Asymmetry	The average difference between two sides in a population sample
**FA**	Fluctuating Asymmetry	Directionally random asymmetry resulting from random perturbations during development
**DI**	Developmental Instability	The inability of an organism to buffer its development against random perturbations
**TA**	Total Asymmetry	Signed difference between the two sides (left-right)
**F-DA**	Fluctuating Directional Asymmetry	Individual variation of asymmetry in the dimension of DA
**C-FA**	Corrected Fluctuating Asymmetry	Fluctuating asymmetry after correcting for DA and F-DA

**Table 2.** Pearson correlation coefficients (r) between the DA vector and the first 5 PCs of the FA vectors, without F-DA correction. The first 5 PCs containing most of the variation in FA show correlation with DA. The statistically significant results are shown in bold (*p* < 0.001).

		PC1	PC2	PC3	PC4	PC5
**Females**	% variance explained	21.7	14.7	11.0	7.0	5.0
	r	**0.08**	**0.36**	**−0.22**	**0.36**	**−0.20**
**Males**	% variance explained	22.1	16.2	12.0	6.8	4.8
	r	**0.40**	**0.57**	0.00	**−0.22**	**0.11**

**Table 3.** Pearson correlation coefficients (r) between the absolute value of F-DA scores with the absolute value of PC scores of C-FA. Although the DA and C-FA vectors are orthogonal (i.e., reflect different dimensions of variation), there is some correlation between their scores for individual faces. Statistically significant results are shown in bold (*p* < 0.001).

		|PC1|	|PC2|	|PC3|	|PC4|	|PC5|
**|F-DA|**	**Females**	**0.14**	**0.19**	**0.17**	**0.22**	**0.15**
	**Males**	0.06	0.08	**0.51**	**0.16**	0.03
